# Multiple Multicolored 3D Polarization Knots Arranged along Light Propagation

**DOI:** 10.1021/acsphotonics.4c01341

**Published:** 2024-09-20

**Authors:** Yan Li, Muhammad Afnan Ansari, Hammad Ahmed, Ruoxing Wang, Guanchao Wang, Qunxing Yu, Chunmei Zhang, Shuqi Chen, Xianzhong Chen

**Affiliations:** † Institute of Photonics and Quantum Sciences, School of Engineering and Physical Sciences, Heriot-Watt University, Edinburgh EH14 4AS, U.K.; ‡ School of Materials, Zhengzhou University of Aeronautics, Zhengzhou 450015, China; § Department of Mathematics and Physics, North China Electric Power University, Baoding 071003, China; ∥ School of Physics, Harbin Institute of Technology, Harbin 150001, China; ⊥ The Key Laboratory of Weak Light Nonlinear Photonics, Ministry of Education, Smart Sensing Interdisciplinary Science Center, Renewable Energy Conversion and Storage Center, School of Physics and TEDA Institute of Applied Physics, Nankai University, Tianjin 300071, China

**Keywords:** optical metasurfaces, polarization knots, metalens, color control, longitudinal control

## Abstract

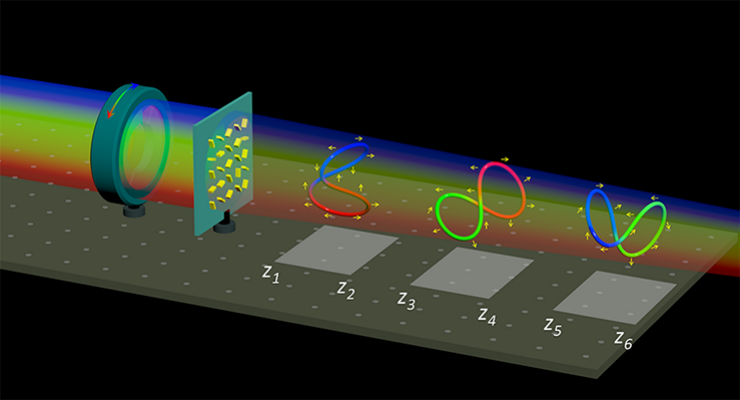

Polarization and color play essential roles in understanding optical phenomena and practical applications. Customized three-dimensional (3D) light fields, characterized by specific polarization and color distributions, have garnered growing interest owing to their unique optical attributes and expanded capacity for information encoding. To align with the ongoing trend of compactness and integration, it is desirable to develop lightweight optical elements that can simultaneously control polarization and color in 3D space. Although engineering longitudinally variable 3D optical structures with predesigned color and polarization information can add more degrees of freedom and additional capacity for information encoding, it has not been reported. We propose a metasurface approach to generating multiple 3D polarization knots along the light propagation direction. Each knot features two colors and an engineered 3D polarization profile. Different multicolored 3D polarization knots are obtained by controlling the observation region along the light propagation. Our approach simultaneously combines polarization, color, and longitudinal control in 3D environment, offering extra degrees of freedom for engineering complex vector beams. The unique properties of the developed metadevices, together with the design flexibility and compactness of metasurface, pave the way for polarization systems with small volumes applicable to some areas such as complex structured beams and encryption.

## Introduction

Polarization and color represent foundational characteristics of light, each serving essential roles in both the comprehension of optical phenomena and their practical applications. 3D polarization profiles are garnering increasing interest owing to their exotic optical characteristics,^1,2^ such as nonorientable surfaces found in Möbius strips,^2,3^ as well as their potential applications, such as lithography^4,5^ and photoalignment for patterning liquid crystals.^6^ Multicolor polarization manipulation will introduce additional degrees of freedom. Achieving color and polarization control in 3D space involves multiple technical challenges, including design complexity, computational methods, wavelength-dependent manipulation, and spatial variation of polarization. Generating multiple multicolored 3D polarization structures lined in a row is both technically and fundamentally challenging, remaining an unanswered question in optics thus far. Moreover, fuelled by miniaturization and integration, there is significant interest in compact optical devices for simultaneously controlling polarization and color in 3D space.

Optical metasurfaces, nanostructured interfaces, have gained big interest for their unparalleled ability to control light propagation at subwavelength scales,^7−17^ revolutionizing optical design with compact platforms for ultrathin optical devices with unusual functionalities. Metalenses are notable examples of how metasurfaces transform traditional optics. The unusual functionalities of metalenses include dual-polarity,^8^ multifoci,^18−20^ polarization rotation,^21^ and compact spectrometers.^22−25^ Recently, a metalens model was used to create 3D polarization structures, including different 3D polarization knots.^26−28^ However, only one color is included in each 3D polarization structure. Multispectral polarization manipulation and longitudinal control will add more degrees of freedom. Additionally, there is a demand for single devices with multiple functionalities in applications prioritizing compactness and integration. Furthermore, the longitudinal control can add additional capacity for information encoding. There exists a question whether we can develop a metalens that encodes color and polarization information into 3D optical structures sequentially along the light propagation direction. However, there have been no reports on generating longitudinally variable 3D optical structures with predesigned color and polarization information embedded within each structure.

To solve the above-mentioned problems, we propose a metasurface approach to performing such a complicated optical task. A single metalens is used to create several muti-colored 3D polarization structures and arrange them in a row along the longitudinal direction. Each knot displays two colors and an engineered 3D polarization profile. Various multicolored 3D polarization knots are obtained by altering the observation zone along the longitudinal direction. Moreover, the polarization structures on these knots can be further modulated by continuously changing the linearly polarized incident light. Polarization knots with different colors can be used to demonstrate longitudinally variable 3D color image steganography, significantly enhancing the information capacity. This approach has addressed the typical challenges for 3D polarization control: multicolor, longitudinal control, and dynamic control. The design flexibility, compactness, and lightweight nature offer numerous desirable features beyond those of conventional polarization optics, which may find applications in many research fields such as complex vector beams generation, color image steganography and 3D display.^29−34^

## Design and Methods

**Figure 1:**
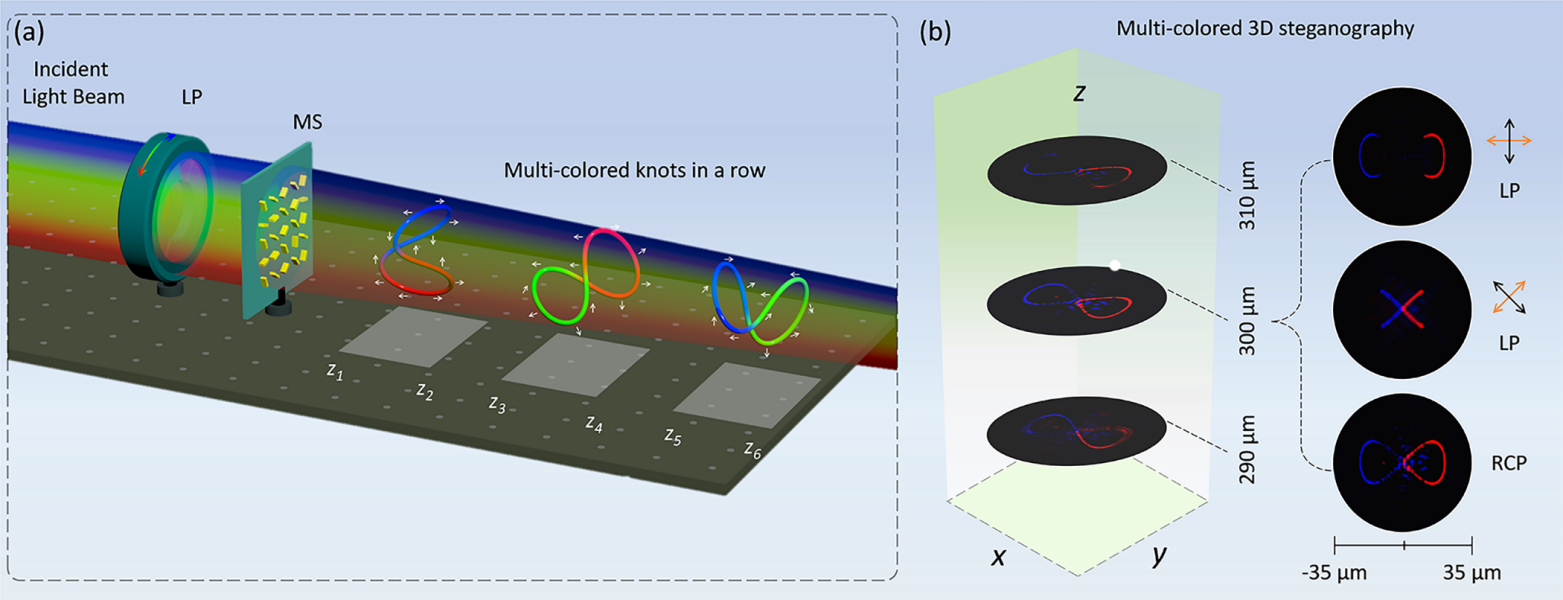
Schematic of the metasurface for creating multicolored 3D polarization knots in a row and realizing multicolor 3D steganography. (a) A single multifoci metalens is used to generate three multicolored 3D polarization structures (2-foil knots with different geometric rotations) that are encoded with different wavelengths and located at three different regions ([*z*_1_, *z*_2_], [*z*_3_, *z*_4_], and [*z*_5_, *z*_6_]) in a row along the light propagation. Different polarization knots can be observed by controlling the incident wavelengths and the observation regions along the *z*-direction. The initial predesigned polarization distributions are depicted in yellow arrows, which can be dynamically modulated by controlling the linear polarization (LP) direction of the incident light. (b) The simulated intensity profiles upon the illumination of an RCP light beam at λ = 650 and 500 nm in the different observation planes of *z* = 290, 300, and 310 μm, respectively (left), which can function as a 3D cover image in 3D steganography. The intensity profiles under the illumination of LP light beam with different polarization directions (orange arrows) after passing through a polarizer (analyzer, black arrows) at the central observation plane of *z* = 300 μm (right). The concealed 3D information can only be revealed with the correct transmission axis of the analyzer and the incident polarization directions.

Figure 1 illustrates the schematic of the proposed metalens designed to generate multiple multicolored 3D polarization knots arranged in a row along the light propagation direction and to realize 3D color steganography. There are two colors involved in each 3D polarization knots. The metalens is comprised of gold nanorods with various orientations on a glass (SiO_2_) substrate. Upon the illumination of incident light with a linear polarization (LP) at three different wavelengths, three distinct 3D polarization knots are generated, each featuring customized colors and unique 3D polarization profiles. Because the three polarization knots are aligned sequentially along the longitudinal direction, different 3D polarization knots can be observed by changing the observation region ([*z*_1_, *z*_2_] for Knot 1, [*z*_3_, *z*_4_] for Knot 2, and [*z*_5_, *z*_6_] for Knot 3) along the longitudinal direction. Additionally, the 3D polarization structures of all these knots (depicted with yellow arrows) can be modulated within the 3D space by merely adjusting the polarization direction of the incident light. The generated 3D polarization structures can be indirectly visualized by observing the modulated intensities, which exhibit distinct features such as dark gaps, after passing through a linear polarizer (analyzer). The modulated 3D intensity patterns, together with color information, represent concealed 3D data, suitable for application in 3D color image steganography. For example, upon the illumination of an RCP light beam at λ = 650 and 500 nm, the intensity profiles in the different observation planes of *z* = 290, 300, and 310 μm are shown in the left panel of Figure 1b, which can function as a 3D cover image in 3D steganography. The different 3D hidden information can be revealed with the correct combination of the transmission axis of the analyzer (black arrows) and the incident polarization directions (orange arrows), as shown in the right panel of Figure 1b. This technique allows for the encoding of diverse 3D hidden color images within the 3D polarization profiles along the longitudinal direction, significantly enhancing the information capacity for encryption purposes.

To generate multiple 3D polarization knots with the predesigned color in a row, the metalens design includes off-axis design, geometric phase realization, linear polarization rotation, multiple 3D knots design, introduction of color information, longitudinal control, and phase multiplexing.

Initially, we consider the phase profile of a metalens with an off-axis focal point based on the Fermat’s principle^8^:
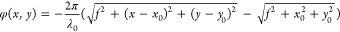
1where (*x*_0_, *y*_0_, *f*) represent the coordinates of the focal point. λ_0_ and *z = f* are the operating wavelength and the focal plane, respectively. To experimentally realize the designed phase profile, we use a plasmonic metasurface with gold nanorods to generate a Pancharatnam-Berry phase profile, whose sign is determined by the helicity of the circularly polarized light, i.e., left circular polarization (LCP) and right circular polarization (RCP).^8,9^ The phase distribution is determined by the orientation angle distribution of gold nanorods.^9^ This is a dual-polarity metalens (convex or concave lens), depending on the helicity of the incident light beam (LCP or RCP).

Apart from the lens focusing functionality, a multifunctional metalens can also realize the polarization rotation by an angle ϕ upon the illumination of the LP light. It is well-known that an LP light beam can be decomposed into LCP and RCP light beams with equal components. The polarization rotation is realized based on the superposition of two circular polarization states with opposite helicity (LCP and RCP).^21^ The designed phase distribution of the metalens for LCP light is given by the formula
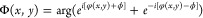
2

A continuous 3D polarization structure is generated by dramatically increasing the number of focal points. The involvement of wavelength information in the design can realize a multicolored 3D polarization knot. Furthermore, the incorporation of the longitudinal coordinate in the design enables the realization of multiple 3D polarization structures at different observation regions. In this design, we need a big number of focal points and independent control of polarization and wavelength at a specific focal point. The designed phase profile for the metalens is governed by
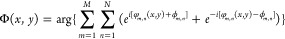
3where
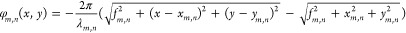
4*M, N,* are the number of polarization knots and total number of points on an individual polarization knot, respectively. *n* and *m* represent the *n*^th^ focal point and the *m*^th^ 3D polarization knot, respectively. λ_*m*,*n*_ is the designed wavelength for the *n*^th^ point on the *m*^th^ 3D polarization knot. (*x*_*m*,*n*_, *y*_*m*,*n*_, *f*_*m*,*n*_) represent the 3D coordinates of an individual focal point whose polarization direction is rotated by an angle ϕ_*m*,*n*_. (*x*, *y*) represent the 2D coordinates of the designed metasurface. The metalens design is based on wavelength and phase multiplexing, which can accurately map the wavelength and polarization information into its focal points located on the knots. The metasurface device is developed by incorporating the intrinsic dispersion and multifoci property of the metalens to realize simultaneous control of wavelength and polarization rotation. The theoretical derivation process is provided in Supplementary Section 1.

## Results

### Two Polarization Knots (ϕ_*m*,*n*_ = α_*m*,*n*_) with Different Colors

**Figure 2:**
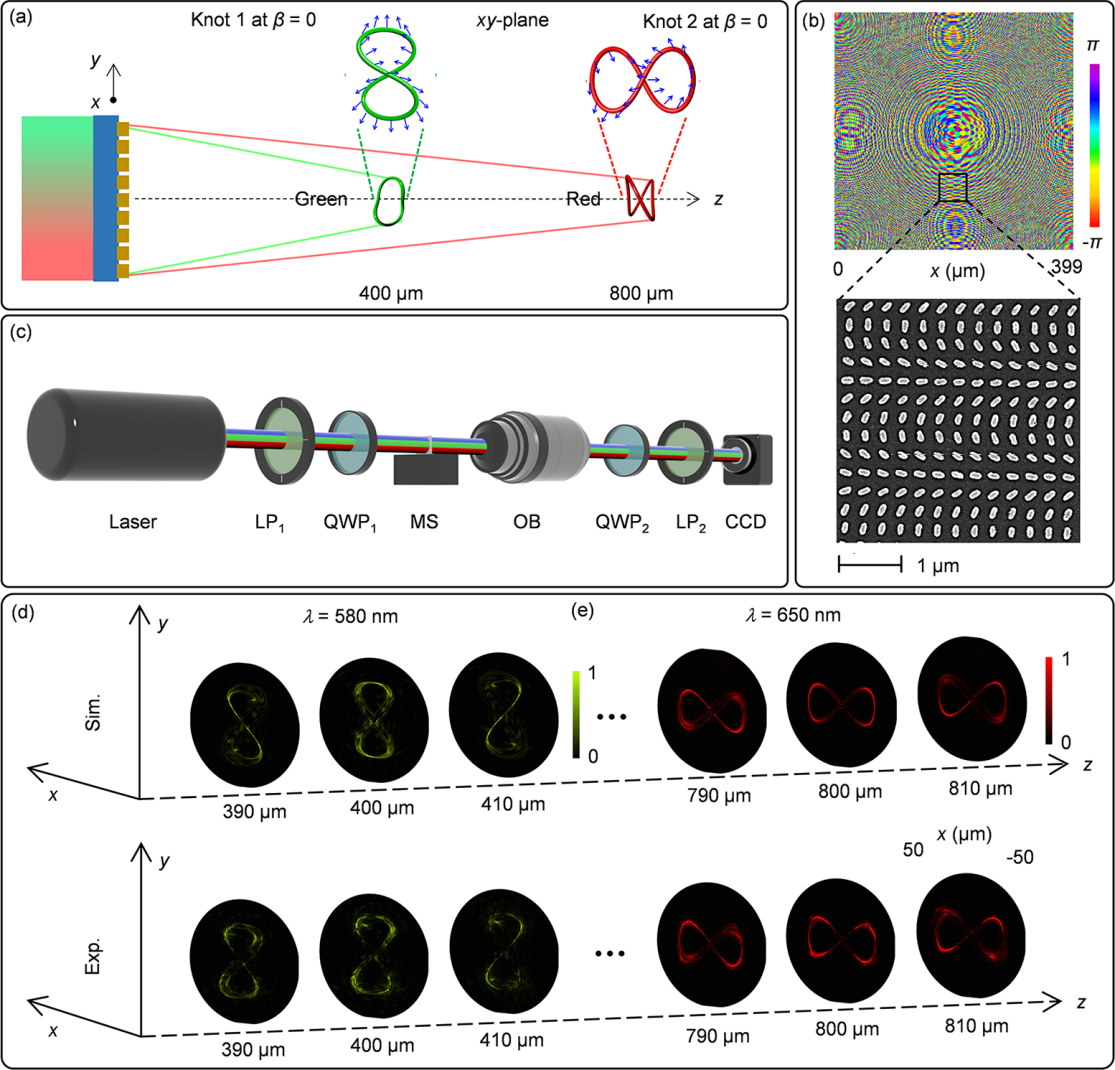
Design principle of two 2-foil 3D knots with different colors in a row, fabricated metasurface and experimental setup. (a) Schematic of the metasurface (MS_1_) design for creating two 2-foil 3D knots encoded with two wavelengths of λ = 580 nm and λ = 650 nm at two predesigned central observation planes of *z* = 400 μm and *z* = 800 μm, respectively. The top panel shows the design of initial polarization profiles on the Knot 1 and Knot 2 in the *xy*-plane under the illumination of LP light with a linear polarization direction along the horizontal direction (β = 0). The polarization directions are depicted with blue arrows. (b) The calculated phase distribution for the MS_1_ with an area of 399 × 399 μm^2^ (top) and the corresponding SEM image (bottom). The scale bar of the SEM image is 1 μm. (c) Schematic of experimental setup for characterizing the fabricated metalens. LP_1_, linear polarizer; QWP_1_ and QWP_2_, quarter-wave plate; LP_2_, linear polarizer (analyzer). LP_1_ and QWP_1_ are used for producing the RCP light. QWP_2_ and LP_2_ are used to filter out the nonconverted part of the output light. The polarization distribution is characterized by removing the two QWPs. A 50× objective (OB) and a charge-coupled device (CCD) camera are used to collect the output light and visualize the 3D knots. The simulated and measured intensity patterns of (d) Knot 1 and (e) Knot 2 at different observation planes under the illumination of RCP light at different wavelengths. The area of the observation plane is 100 × 100 μm^2^.

To verify the design strategy, we start with a multifoci metalens with a diameter of 399 μm to generate two 2-foil polarization knots with different colors along the light propagation direction. The spatial coordinates of an individual focal point on the 2-foil polarization knots are governed by
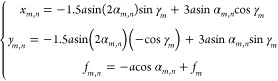
5where the parameter *a* is set as 10 μm, which controls the size of the knots. The polar angle α_*m*,*n*_ ranges from 0 to 2π. γ_*m*_ is the geometric rotation angle of the knots, which are π/2 for Knot 1 (*m* = 1) and 0 for Knot 2 (*m* = 2), respectively. The *f*_*m*_ defines the central focal plane of Knot *m*. Here, the coordinates of the central focal planes along the longitudinal direction for Knot 1 and Knot 2 are set as *z* = 400 and 800 μm, respectively. The numbers of focal points for Knot 1 and Knot 2 are 1000 and 700, respectively. All the focal points on each knot are designed with a single wavelength λ_*m*,*n*_ (λ_1,*n*_ = 580 nm (green) for the Knot 1 and λ_2,*n*_ = 650 nm (red) for the Knot 2, respectively). The polarization distribution ϕ_*m*,*n*_ of the *n*^th^ focal point on the Knot *m* is initially set as ϕ_*m*,*n*_ = α_*m*,*n*_. Two 3D knots with customized colors and polarization profiles are generated in a row along the light propagation (*z*-direction) with the customized central focal planes (see Figure 2a).

A plasmonic metasurface consisting of nanorods is used to realize the designed metadevice. Each nanorod is 40 nm thick, 80 nm wide, and 200 nm long. The pixel size is 300 nm × 300 nm. The unit cell design details and the efficiency analysis of metasurfaces are given in Supplementary Section 2. The fabrication details of metasurfaces are available in Experimental Section. The calculated phase profile of the metasurface and the scanning electron microscopy (SEM) image of the fabricated sample are given in Figure 2b. The optical setup for metalens characterize is given in Figure 2c. A supercontinuum laser source (NKT Photonics SuperK EXTREME) is used to generate an incident light beam with tunable wavelengths. The 3D polarization structures are revealed with an analyzer LP_2_ upon the illumination of incident LP light. Detailed experimental design is given both in Experimental Section and in Supplementary Section 3. Here, all the simulated intensity patterns of the 3D knots are obtained based on the Fresnel–Kirchhoff diffraction integral.^35^

To characterize the change of light intensity profiles and colors of two 3D polarization knots, we choose six different observation planes along the longitudinal direction and employ two different operating wavelengths in the simulation and experimental measurement. Figure 2d,e shows the simulation (top) and experimental results (bottom) under the illumination of RCP light beam at λ = 580 nm for Knot 1 (Figure 2d) and at λ = 650 nm for Knot 2 (Figure 2e), respectively. The three observation planes for Knot 1 are located at *z* = 390, 400, and 410 μm, respectively, while that for Knot 2 are located at *z* = 790, 800 and 810 μm, respectively. Two 3D structures with different colors are simultaneously generated at different regions along the light propagation (*z*-direction). The metasurface is also illuminated by the RCP light beams with two wavelengths of 580 and 650 nm, simultaneously. The corresponding simulated and measured intensity distributions at six different observation planes are presented in Supplementary Section 4.1. As shown in Figures 2d,e and S2, the change in intensity profiles and colors of the designed 3D structures along the longitudinal direction clearly demonstrates longitudinally variable and color-controllable 3D nature of the polarization knots.

**Figure 3:**
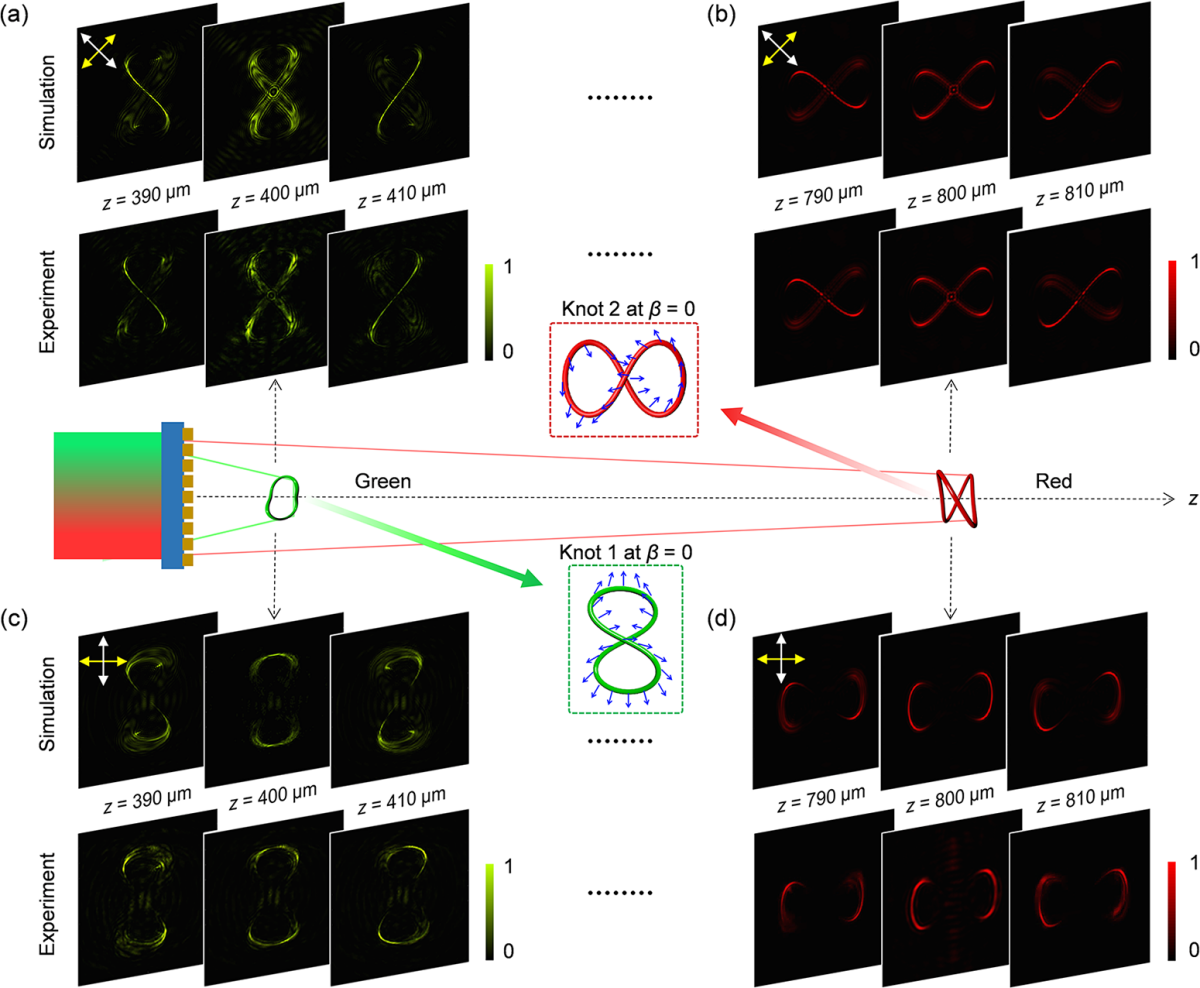
Dynamic control and characterization of the 3D polarization profiles on two 2-foil knots at λ = 580 nm and λ = 650 nm along the longitudinal direction. To confirm the 3D polarization profiles on each knot, we provide the simulated (top) and measured (bottom) intensity profiles at three different observation planes (*z* = 390, 400 and 410 μm for Knot 1, and *z* = 790, 800 and 810 μm for Knot 2) after light passes through the analyzer LP_2_. Yellow and white arrows represent the transmission axes of LP_1_ and LP_2_, which are perpendicular to each other in the case of LP incidence. The simulation and experimental results of the (a) Knot 1 at λ = 580 nm and (b) Knot 2 at λ = 650 nm upon the illumination of incident light with a linear polarization along the diagonal direction (β = π/4). (c) and (d) The corresponding results when the linear polarization direction of incident light is changed from the diagonal direction (β = π/4) to the horizontal direction (β = 0).

To reveal the created 3D polarization structures for the incident LP light, the two QWPs in the optical setup (Figure 2c) are removed. The transmission axes of two polarizers (LP_1_ and LP_2_) are maintained orthogonal to each other, which can filter out the nonconverted part. In addition, this setup can confirm the polarization nature of the 3D knots through the modulated intensity distribution governed by the Malus’ law (see Supplementary Section 3). The simulated (top) and measured (bottom) intensity distributions shown in Figure 3 are the results under the illumination of LP light at λ = 580 nm for Knot 1 and at λ = 650 nm for Knot 2, respectively. Different combinations of the transmission axes of LP_1_ and LP_2_ are analyzed. It is noteworthy that the intensity minimum corresponds to the dark position where the designed polarization rotation angle ϕ equal to 2β and 2β + π (β is the incident linear polarization direction with respect to the *x* axis). Therefore, the dark gaps in the intensity patterns are dynamically modified by simultaneously rotating LP_1_ and LP_2_ and maintaining perpendicularly between the two transmission axes. For example, for β = π/4, dark gaps can be clearly observed at the locations with ϕ = π/2 and 3π/2 (see Knot 1 in Figure 3a and Knot 2 in Figure 3b). Similarly, for β = 0, dark gaps appear at ϕ = 0 and π (see Knot 1 in Figure 3c and Knot 2 in Figure 3d). Detailed simulation and measurement results for the incident LP light with two operating wavelengths of 580 and 650 nm are provided in Supplementary Section 4.2. The strong agreement between simulation and experimental measurements unequivocally demonstrates the dynamic control of 3D polarization structures, enabling the creation of predetermined colors along the path of light propagation.

### Three 2-Color Polarization Knots (ϕ_*m*,*n*_ ≠ α_*m*,*n*_)

**Figure 4:**
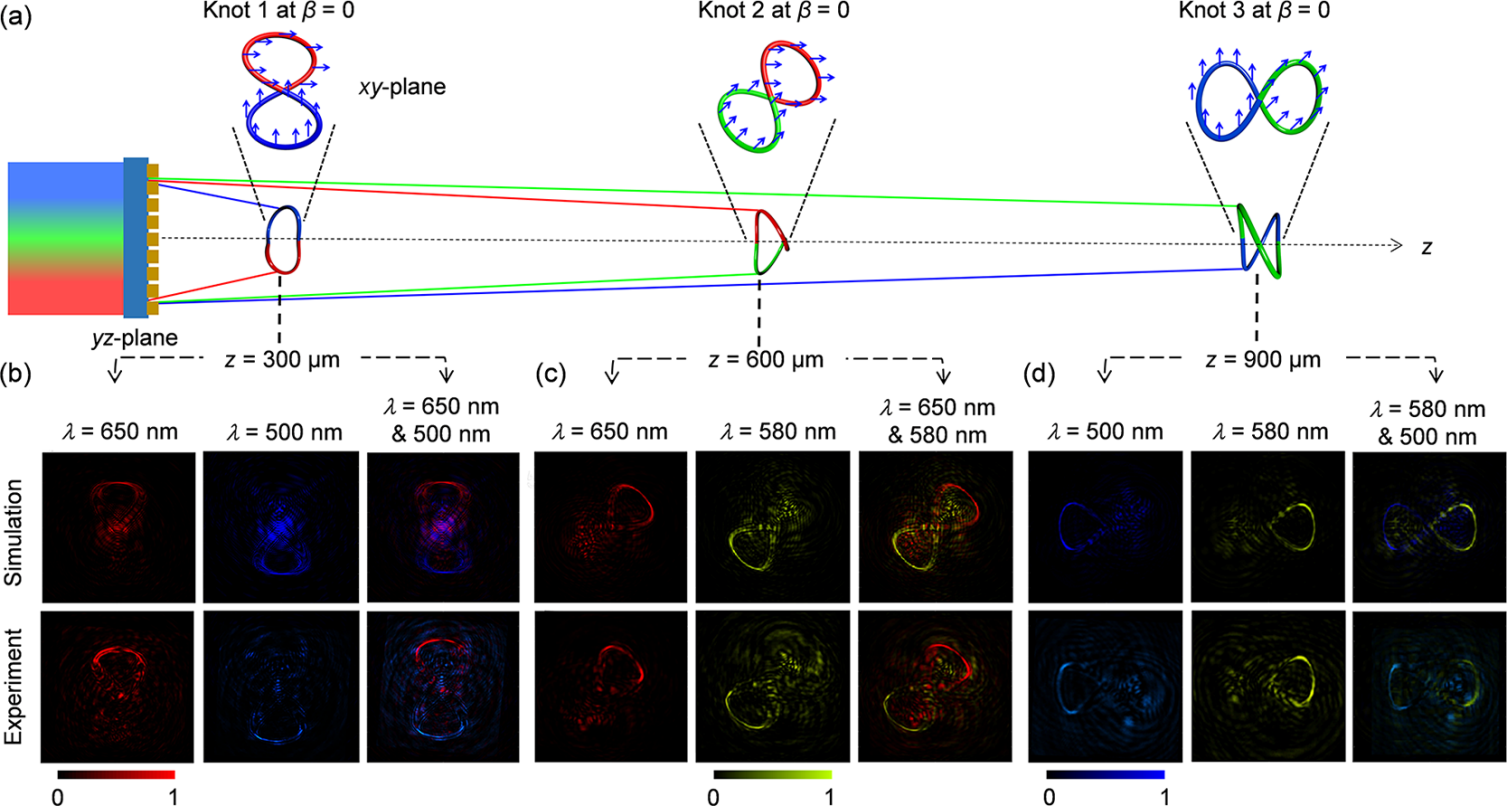
Metasurface for creating three multicolored 3D polarization knots along the longitudinal direction. Second metasurface (MS_2_) is designed to produce three 2-foil 3D knots whose central observation planes are located at *z* = 300, 600 and 900 μm, respectively. (a) The design of the color and initial polarization distribution on the Knots 1–3 in *xy*-plane under LP light beam with a polarization direction β = 0. The red, green, blue colors on three 2-foil knots represent the encoded wavelengths λ = 650, 580 and 500 nm, where the corresponding polarization rotation angles are set as 0, π/4 and π/2 denoted with blue arrows. (b–d) Light intensity distributions for (b) Knot 1, (c) Knot 2, and (d) Knot 3 under the single- and two-wavelength illumination of RCP light beam.

In the above examples, we use the proposed method to create two polarization knots encoded with two different wavelengths and dynamically control them at a pixel level. However, for certain applications requiring high information capacity, such as information security and anticounterfeiting, having more than two multicolored 3D polarization structures in a row is desirable. Generating three multicolored 3D polarization structures in a row along the longitudinal direction (shown in Figure 4a) has demonstrated the capability of the proposed approach with the increased information capacity. Equation 5 is used to design each 2-foil knot as show in Figure 4a (top). Here, the geometric rotation angles γ_*m*_ of the three 2-foil polarization knots are π/2 (*m* = 1 for Knot 1), π/4 (*m* = 2 for Knot 2), and 0 (*m* = 3 for Knot 3), respectively. The three knots have three different central observation planes, which are located at *z* = 300, 600, and 900 μm, respectively. The numbers of focal points (denoted by *N*) on the three polarization knots are 1000, 750 and 600, respectively. The focal points ranging from 1 to 500 on Knot 1 and focal points 1 to 375 on Knot 2 are encoded with a wavelength of 650 nm, where the polarization rotation distribution is set as ϕ_1,*n*_ = ϕ_2,*n*_ = 0. Moving to the next set of focal points, specifically focal points 501 to 1000 on Knot 1 and focal points 301 to 600 on Knot 3, they are encoded with a wavelength of 500 nm, where the predesigned polarization profile is set as ϕ_1,*n*_ = ϕ_3,*n*_ = π/2. Finally, the focal points ranging from 376 to 750 on Knot 2 and focal points 1 to 300 on Knot 3 are encoded with the wavelength of 580 nm, where the polarization rotation distribution is set as ϕ_2,*n*_ = ϕ_3,*n*_ = π/4. The initial polarization distributions of these 2-color knots are denoted with blue arrows in the *xy*-plane in Figure 4a (top).

The simulated (top) and measured (bottom) results for the incident RCP light at both single and dual wavelengths are presented at three central observation planes of three knots *z* = 300 μm (Figure 4b), 600 μm (Figure 4c), and 900 μm (Figure 4d). The results validate the presence of three multicolored 3D structures. When the metalens is illuminated by RCP light at single wavelength (λ = 650 nm), the red foils of Knot 1 and Knot 2 are observed at the central observation planes of *z* = 300 and 600 μm (the first column in Figure 4b,c). By tuning the operating wavelength to 580 and 500 nm (λ = 580 nm for the second column in Figure 4c,d, and λ = 500 nm for the second column in Figure 4b and the first column in Figure 4d), the blue and green foils on the Knots 1–3 are obtained, respectively. Under dual-wavelength illumination, the complete red-blue Knot 1, red-green Knot 2 and blue-green Knot 3 can be found on the three observation planes (the third column in Figure 4b–d). Therefore, different portions of each knot can be obtained by changing the incident wavelength, which clearly shown the color-encoding functionality of the designed metalens.

**Figure 5:**
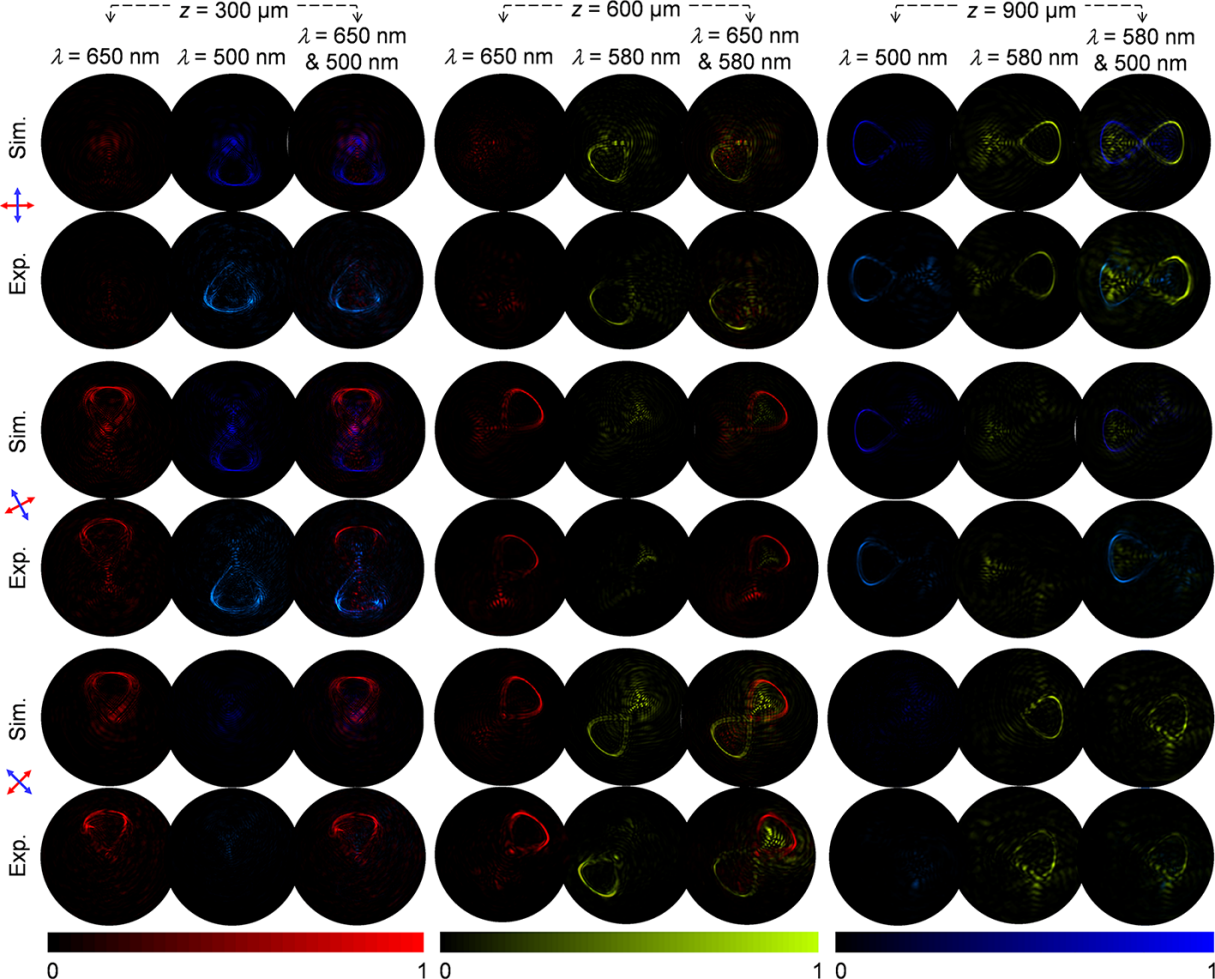
Dynamic control of three 2-color 3D polarization knots. (a–c) The simulated and measured intensity distributions of (a) Knot 1, (b) Knot 2, and (c) Knot 3 at their corresponding central observation planes (*z* = 300, 600, and 900 μm) under the single- and dual-wavelength illumination with different polarization states of LP incident light beam. Red and blue arrows show the directions of transmission axes of the LP_1_ and LP_2_, which are perpendicular to each other.

Dynamic control of 3D polarization profiles is demonstrated by controlling the incident linear polarization. Figure 5a–c shows the light intensity and polarization distributions at the predesigned central observation planes for the Knots 1–3, respectively. When LP_1_ and LP_2_ have their transmission axes fixed at (0, π/2), (π/8, 5π/8), and (π/4, 3π/4), the corresponding red foils of Knots 1 and 2 with the initial polarization rotation angle ϕ = 0 (Columns 1 and 3 of Rows 1 and 2 in Figure 5a,b), green foils of Knots 2 and 3 with ϕ = π/4 (Columns 2 and 3 of Rows 3 and 4 in Figure 5b,c), and blue foils of Knots 1 and 3 with ϕ = π/2 (Columns 2 and 3 of Rows 5 and 6 in Figure 5a and Columns 1 and 3 of the Rows 5 and 6 in Figure 5c) disappear based on the Malus’ law, respectively. The measured results at nine different observation planes under two-wavelength illumination further validate the existence of three multicolored 3D polarization knots in a row along the longitudinal (details provided in Supplementary Section 5).

## Discussion

The light intensity distributions of the 3D polarization knots are modulated with a rotating analyzer, generating unusual features (e.g., dark gaps). Such a characteristic opens an avenue for image steganography—a technique which can hide a secret image inside a cover image. In our design, although the 3D polarization knots have fixed shapes, we can encode different combinations of polarization states and colors for the focal points on the 3D knots. Furthermore, when the linear polarization direction of the incident light is changed, the linear polarization state at each focal point can be rotated, dynamically modulating the 3D multispectral intensity patterns with unique features (e.g., dark gaps) in the observation regions when passing through a linear polarizer (analyzer). These modulated intensity patterns with color information can be used for 3D color image steganography. A polarization knot with the same light intensity profile can have many 3D polarization distributions and colors. The light intensity pattern of the color knots can work a 3D cover image, while the intensity patterns after modulation based on the Malus’ law can be used to conceal various secret 3D color images. Our proposed metalens can realize multiple 3D color images steganography along the longitudinal direction, dramatically enhancing the information capacity. The hidden 3D color information can only be decoded with the correct combination of the operating wavelength, transmission axes of the polarizer and analyzer, and the longitudinal coordinate (*z*).

**Figure 6:**
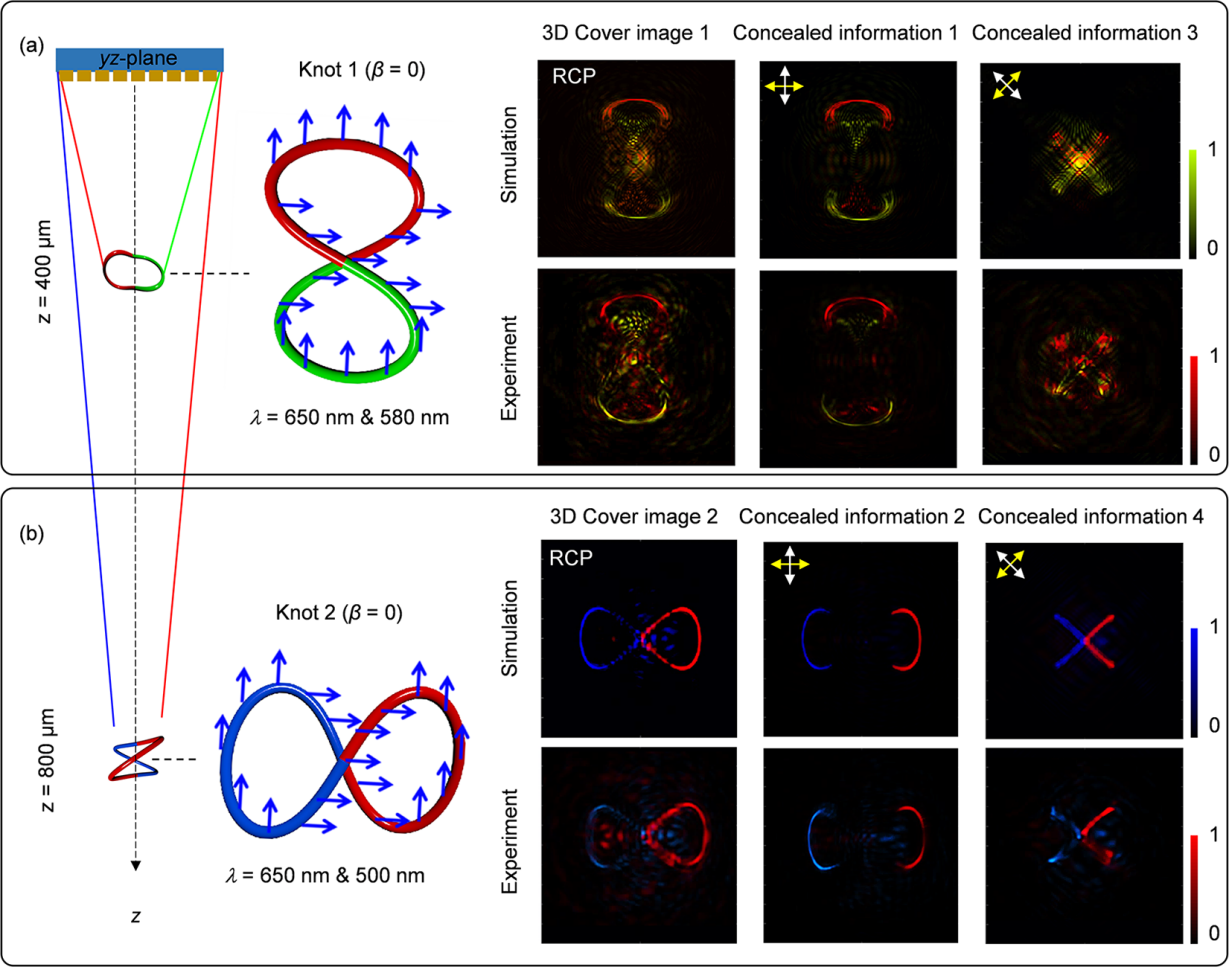
Polarization knots for longitudinally variable 3D color image steganography. The intensity pattern of the color knots can function as a 3D cover image, while the modulated intensity patterns based on the Malus’ law can be used to hide many secret 3D color images. The concealed 3D color information can only be revealed with the correct operating wavelength, transmission axes of the polarizer and analyzer, and the longitudinal coordinate (*z*). (a) Schematic of the 3D cover image 1 at its central observation plane of *z* = 400 μm (left panel), simulated and measured cover image 1 under the illumination of RCP light (middle panel), and two different revealed hidden 3D images inside cover image 1 under special polarizer–analyzer combinations in the case of LP incidence (right panel). (b) Another 3D image steganography at its central observation plane of *z* = 800 μm. The concealed 3D information here is the portions of 2-foil 3D knots and located in 3D space.

The concealed 3D information is the part of 2-foil 3D polarization knots. To demonstrate the longitudinally variable 3D color image steganography technique, two multicolored polarization knots located at *z* = 400 and 800 μm are designed, as depicted in the left panels in Figure 6a,b. Specifically, the Knot 1 (*m* = 1) is encoded with wavelengths of 650 and 580 nm, whereas the Knot 2 (*m* = 2) are designed with wavelengths of 650 and 500 nm. The polarization rotation angles for Knot 1 are set as ϕ_1*n*_ = 0 for | *y*_1*n*_ | < 15 μm, and ϕ_1*n*_ = π/2 for |*y*_1*n*_ | ≥ 15 μm. Regarding Knot 2, polarization rotation angles are set as ϕ_2*n*_ = 0 for |*x*_2*n*_ | < 15 μm and ϕ_2*n*_ = π/2 for |*x*_2*n*_ | ≥ 15 μm. The intensity profiles of the two polarization knots under the illumination of RCP light with dual operating wavelengths are used as the 3D cover images (Figure 6a,b). Simulated and measured light intensity patterns of 3D cover images at different observation planes are presented in Supplementary Section 6. The modulated 3D intensity patterns can be considered as 3D hidden information. For instance, the central portions of the 2-foil knots (|*y*_1*n*_ | < 15 μm for Knot 1 and |*x*_2*n*_ | < 15 μm for Knot 2) are hidden when the transmission axes of the LP_1_ and LP_2_ are 0 and π/2, respectively. When the transmission axes of the LP_1_ and LP_2_ are rotated as π/4 and 3π/4, the central portions of the 2-foil knots are clear but the other portions (|*y*_1*n*_ | ≥ 15 μm for Knot 1 and |*x*_2*n*_ | ≥ 15 μm for Knot 2) are hidden. Furthermore, the desired concealed 3D information can be revealed at the predesigned observation areas under the illumination of an incident LP light beam at predesigned wavelengths and with correct transmission axes of the polarizer LP_1_ and analyzer LP_2_. Therefore, different combinations of the transmission axis of the LP_1_ (yellow arrows) and LP_2_ (white arrows), incident wavelengths and the longitudinal position (*z*) can function as different keys to reveal multiple distinct concealed 3D information. For instance, the concealed 3D information 1, as shown in Figure 6a, can be obtained with correct key combination i.e., the transmission axes of the LP_1_ and LP_2_ (0 and π/2), incident wavelengths (λ = 650 and 580 nm) and longitudinal positions (*z* = 300 μm), respectively. The light intensity patterns with hidden 3D information at various observation planes for the transmission axes of the LP_1_ and LP_2_ set as 0 (π/4) and π/2 (3π/4) are presented in Supplementary Section 6. It is worth mentioning that the intensity patterns on the observation planes are affected by the crosstalk between different colors in the same polarization knots and that between different polarization knots (details provided in Supplementary Section 7.1). Additionally, the crosstalk for the Knot 1 at *z* = 400 μm is stronger than that for the Knot 2 at *z* = 800 μm (details provided in Supplementary Section 7.2). Potential solutions to decrease crosstalk include increasing the encoded wavelength interval for each knot, reducing the longitudinal size of the knots and increasing the area of metasurface. Figures S9 and S10 show that the parasitic light can be effectively suppressed, and higher-quality color knots can be achieved by increasing the encoded wavelength interval (Figure S9) and increasing the metasurface area while reducing the longitudinal size of the polarization knot (Figure S10). It is clearly shown that the longitudinally variable 3D color image steganography is demonstrated based on the modulated light intensity distributions in the decoding process. Unlike image steganography technology with a single color, our method can dramatically increase the encryption capacity due to the color involvement.

We present the first experimental demonstration of creating multicolored 3D polarization knots in a row along the light propagation direction based on a multifoci metalens. These 3D polarization knots can be dynamically modulated by changing the linear polarization direction and wavelength of the incident light. Unlike our previous works,^26−28^ where only a single wavelength and a single-color are included in the 3D polarization structures, multiple wavelengths and 3D polarization structures with various colors are included here. The color-encoding property is realized through dispersion effect and the unique design of the metasurface. This novel design allows for the realization of different 3D polarization structures at different observation regions along the light propagation by changing the incident wavelength. In this work, the maximum number of wavelengths encoded in each 3D polarization structure is two, which can be increased to further expand the information capacity. The simulated intensity distributions of two 2-foil 3D polarization knots (each knot is encoded with three wavelengths of 650, 580 and 500 nm) under different incident polarization states are provided in Supplementary Section 10, respectively. The image quality of the color polarization knots on the observation planes decreases as the number of encoded wavelengths per knot increases due to the more crosstalk between different colors. One possible solution to improve the image quality is to enlarge the metasurface area while simultaneously reducing the longitudinal size of the polarization knots. Additionally, increasing the encoded wavelength interval can increase dispersion and then suppress the crosstalk. Simultaneously encoding color and polarization information into 3D structures along the light propagation can realize multiple 3D color images concealed in multiple 3D color images along the light propagation. The decoded color image in the modulated intensity profile with the customized color can be switched to other color image (encoded image), which is governed by the Malus’ law with the assistance of an analyzer (polarizer). Simulation and experimental results in Figure 6 showcase the longitudinally variable 3D color image steganography.

## Conclusions

In summary, we develop a metalens that can generate several multicolored polarization knots and arrange them in a row along the light propagation direction. Our work has solved several typical issues with 3D polarization generation: design flexibility, color control and longitudinal control, offering more degrees of freedom for simultaneous control of polarization and color in 3D space. As a proof-of-concept demonstration, we use a metalens to realize multiple 3D color images hidden in multiple 3D color images along the light propagation. This work lays the foundation for lightweight polarization systems applicable to areas such as complex structured beams and encryption.

## Experimental Details

### Sample Fabrication

We use plasmonic metasurfaces consisting of gold nanorods with various orientations on an ITO-coated glass substrate, which are fabricated with electron beam lithography, film deposition, and a lift-off process. An individual metasurface has an area of 399 × 399 μm^2^. First, an ultrasonic bath is used to clean the glass substrate with acetone for 10 min, followed by isopropyl alcohol (IPA) for another 10 min. Subsequently, we use a 100 nm-thick film of poly(methyl methacrylate) (PMMA) 950 A2 on the substrate by spin coating at 1000 rpm for 60 s. Then, a hot plate is used to bake the sample at 180 °C for 5 min. The electron beam lithography (Raith PIONEER, 12 pA, 30 kV) is used for nanopatterning. After the electron beam exposure, the sample is developed in the mixture of MIBK:IPA (1:3) for 45 s, and then rinsed with IPA for 45 s. An electron beam evaporator is used to deposit a 40 nm-thick gold film on the sample. Finally, the metasurface is ready for characterization after the lift-off process in acetone for 10 h.

### Experimental Setup

Figure 2c shows the experimental setup for characterizing the fabricated metasurfaces. A supercontinuum laser (NKT Photonics SuperK EXTREME) is used to generate an incident light beam with tunable wavelengths. The polarization state of the incident light is controlled with a linear polarizer (LP_1_) and a quarter wave plate (QWP_1_). For example, RCP light is generated if the angle between the transmission axis of LP_1_ and the fast axis of QWP_1_ is π/4. An objective with a magnification of 50× and a charge-coupled device camera (CCD) are used to collect the output light on the transmission side and capture the 3D polarization knots for visualization. To observe the light intensity patterns of 3D knots at different observation planes along the light propagation (*z* direction), the objective lens is mounted on a motorized translation stage. Another pair of a quarter wave plate (QWP_2_) and a linear polarizer (LP_2_) after the objective is used to remove the nonconverted part. To evaluate the performance of the developed metadevice for the incident linear polarization, both QWP_1_ and QWP_2_ are removed and the transmission axes of LP_1_ and LP_2_ are perpendicular to each other. The experimental setup is also explained in Supplementary Section 3.

## Supporting Information Available

Working principle of metalens with polarization rotation functionality, unit cell design and conversion efficiency of metasurfaces, working principle of the experimental setup, simulations and experiments of two 2-foil knots (ϕ_*m*,*n*_ = α_*m*,*n*_) with two operation wavelengths, three 2-foil polarization knots (ϕ_*m*,*n*_ ≠ α_*m*,*n*_) with three wavelengths, and longitudinally variable 3D color image steganography, creating two 2-foil knots (ϕ_*m*,*n*_ ≠ α_*m*,*n*_) with three colors along the longitudinal direction (PDF)
