# Supplementary material for: Multiple Multicolored 3D Polarization Knots Arranged along Light Propagation
Source: ACS Photonics. 2024 Sep 20;11(10):4380–9. doi: 10.1021/acsphotonics.4c01341 (PMC11487683; doi:10.1021/acsphotonics.4c01341)
Supplement: Supplementary file 1 — ph4c01341_si_001.pdf [file ph4c01341_si_001.pdf]

Supporting information for

Multiple multi-colored 3D polarization knots arranged along light propagation

Yan Li[a,b,†], *Muhammad Afnan Ansari*[a,†], *Hammad Ahmed*[a], *Ruoxing Wang*[c], *Guanchao Wang*[a,d], *Qunxing Yu*[b], *Chunmei Zhang,*[a] *Shuqi Chen,*[e] *and Xianzhong Chen*[a,*]

[a]Institute of Photonics and Quantum Sciences, School of Engineering and Physical Sciences, Heriot-Watt University, Edinburgh, EH14 4AS, UK

[b]School of Materials, Zhengzhou University of Aeronautics, Zhengzhou 450015, China

[c]Department of Mathematics and Physics, North China Electric Power University, Baoding 071003, China

[d]School of Physics, Harbin Institute of Technology, Harbin 150001, China

[e]The Key Laboratory of Weak Light Nonlinear Photonics, Ministry of Education, Smart Sensing Interdisciplinary Science Center, Renewable Energy Conversion and Storage Center, School of Physics and TEDA Institute of Applied Physics, Nankai University, Tianjin 300071, China

This Supporting Information contains:

Number of pages: 19

Number of figures: 11

Number of tables: 0

Supplementary Section 1. Working principle of metalens with polarization rotation functionality

To design a metalens with an arbitrary continuous focal structure with color information in 3D space, we first formulate the phase distribution of the lens with an off-axis focal point, which is given by:

$$\varphi(x,y) = -\frac{2\pi}{\lambda_0}\left(\sqrt{f^2 + (x - x_0)^2 + (y - y_0)^2} - \sqrt{f^2 + x_0^2 + y_0^2}\right). \quad \text{(S1)}$$

where (x_0, y_0, f) represent the coordinates of the focal point. λ_0 and $z = f$ are the operating wavelength and the focal plane, respectively, and $\sqrt{f^2 + x_0^2 + y_0^2}$ represents the distance between the focal point and center of the metalens. (x, y) are coordinates of the metasurface. Eq. (S1) is used for focusing one circularly polarized (CP) light beam. The desired phase profile for focusing a linearly polarized (LP) light beam is governed by Eq. (S2):

$$\Phi(x,y) = \arg(e^{i\varphi(x,y)} + e^{-i\varphi(x,y)}). \quad \text{(S2)}$$

where the term $e^{-i\varphi_n(x,y)}$ is added to focus a right circularly polarized (RCP) light beam and the term $e^{i\varphi_n(x,y)}$ in Eq. (S2) is responsible for focusing the left circularly polarized (LCP) light beam. To realize polarization rotation, the required phase distribution for such metadevice is governed by Eq. (S3):

$$\Phi(x,y) = \arg(e^{i[\varphi(x,y)+\phi]} + e^{-i[\varphi(x,y)-\phi]}). \quad \text{(S3)}$$

where ϕ is the polarization rotation angle at the focal point. The resultant electric field is comprised of four different components which possess distinct phases, i.e., $E_L e^{-i[\varphi(x,y)+\phi]}$, $E_L e^{i[\varphi(x,y)-\phi]}$, $E_R e^{-i[\varphi(x,y)-\phi]}$, and $E_R e^{i[\varphi(x,y)+\phi]}$. Where E_L and E_R are the amplitudes of LCP and RCP light beams. Among these four CP components, only $E_L e^{i[\varphi(x,y)-\phi]}$ and

$E_R e^{i[\varphi(x,y)+\phi]}$ are responsible for the construction of desired polarization rotation of each focal point.

By increasing the number of focal points in 3D space, the phase profile of a 3D structure (continuous focal curve) can be expressed as:

$$\Phi(x,y) = \arg\left\{\sum_{n=1}^{N} e^{i[\varphi_n(x,y)+\phi_n]} + e^{-i[\varphi_n(x,y)-\phi_n]}\right\}. \tag{S4}$$

where N represents the total number of points on a specific 3D structure. To convert a LP light beam into longitudinally variable 3D polarization knots with color information. The desired phase profile for the metalens upon the illumination of the incident RCP light is given by:

$$\Phi(x,y) = \arg\left\{\sum_{m=1}^{M}\sum_{n=1}^{N}\left(e^{i[\varphi_{m,n}(x,y)+\phi_{m,n}]} + e^{-i[\varphi_{m,n}(x,y)-\phi_{m,n}]}\right)\right\}\quad , \tag{S5}$$

where

$$\varphi_{m,n}(x,\ y) = -\frac{2\pi}{\lambda_{m,n}}\left(\sqrt{f_{m,n}^2 + (x - x_{m,n})^2 + (y - y_{m,n})^2} - \sqrt{f_{m,n}^2 + x_{m,n}^2 + y_{m,n}^2}\right). \tag{S6}$$

M and m represent the total number of 3D polarization structures and the mth polarization structure, respectively. $\lambda_{m,n}$ is the wavelength for the nth focal point on the mth structure. $(x_{m,n}, y_{m,n}, f_{m,n})$ represent the coordinates of a given point with a polarization rotation angle of $\phi_{m,n}$ on created 3D polarization structures.

Supplementary Section 2. Unit cell design and conversion efficiency of metasurfaces

The calculated conversion efficiency is relatively flat and uniform within the design band ranging from 480 nm to 700 nm, with a conversion efficiency exceeding 8%, as shown in **Figure S1b**. The intensity of the converted part is lower than that of the non-converted part, which can be filtered out by using the experimental setup in **Figure 2c**.

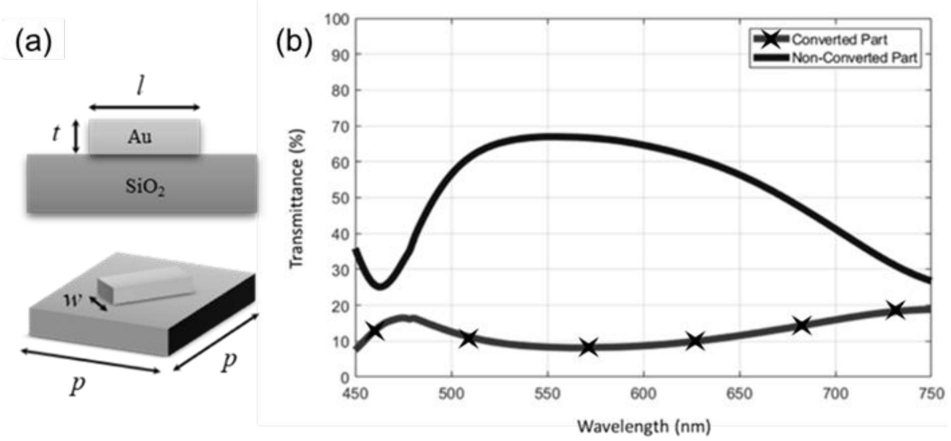

Figure S1. Transmission efficiency of the metasurface. (a) Unit cell design. (b) Non-converted and converted components of the transmission spectra in the whole visible region. The width $w = 80$ nm, length $l = 200$ nm, thickness $t = 40$ nm and pixel size $p = 300$ nm.

Supplementary Section 3. Working principle of the experimental setup

The Jones vectors and Jones matrices are used to represent the polarization states of the light beams and the functionality of the optical elements. Each nanorod in the plasmonic metasurface can be considered as the combination of a perfect polarizer (low percentage) and a piece of normal flat glass slab, $\begin{bmatrix} 1 & 0 \\ 0 & 1 \end{bmatrix}$. Thus, the Jones matrix of each nanorod $(J_{nanorod})$ can be written as:

$$J_{nanorod} = A\begin{bmatrix} 1 & 0 \\ 0 & 1 \end{bmatrix} + B\begin{bmatrix} \cos^2\theta & \sin\theta\cos\theta \\ \sin\theta\cos\theta & \sin^2\theta \end{bmatrix}. \qquad \text{(S7)}$$

where θ is an orientation angle of each nanorod with respect to the x axis. A and B are the coefficients of the conversion efficiency of that glass slab and nanorods, respectively. In **Figure 2c** in the main text, the incident light beam (E_{in}) is generated by the polarizer LP_1 with the transmission axis along the x direction. Thus, its Jones vector is given by $E_{in} = \begin{bmatrix} 1 \\ 0 \end{bmatrix}$. The

polarization state of the E_{in} can be changed by using the quarter waveplate QWP_1, whose Jones matrix is given by:

$$J_{QWP1} = e^{-\frac{i\pi}{4}}\begin{bmatrix} \cos^2\chi_1 + i\sin^2\chi_1 & (1-i)\sin\chi_1\cos\chi_1 \\ (1-i)\sin\chi_1\cos\chi_1 & \sin^2\chi_1 + i\cos^2\chi_1 \end{bmatrix}. \quad \text{(S8)}$$

where χ_1 is the orientation angle of the fast axis with respect to the x-direction. When the E_{in} passes through the QWP_1 with the angle χ_1 of $\frac{\pi}{4}$, the output vector can be calculated by:

$$J_{QWP1}E_{in} = e^{-\frac{i\pi}{4}}\begin{bmatrix} \cos^2\frac{\pi}{4} + i\sin^2\frac{\pi}{4} & (1-i)\sin\frac{\pi}{4}\cos\frac{\pi}{4} \\ (1-i)\sin\frac{\pi}{4}\cos\frac{\pi}{4} & \sin^2\frac{\pi}{4} + i\cos^2\frac{\pi}{4} \end{bmatrix}\begin{bmatrix} 1 \\ 0 \end{bmatrix} = \frac{1}{\sqrt{2}}\begin{bmatrix} 1 \\ -i \end{bmatrix}. \quad \text{(S9)}$$

Here, an RCP light beam is generated. When the RCP beam passes through a nanorod, the Jones vector of the transmitted beam can be written as:

$$J_{nanorod}RCP = \frac{2A+B}{2\sqrt{2}}\begin{bmatrix} 1 \\ -i \end{bmatrix} + \frac{B}{2\sqrt{2}}e^{-i2\theta}\begin{bmatrix} 1 \\ i \end{bmatrix}. \quad \text{(S10)}$$

The transmitted light includes two main parts, non-converted part: $E_{nc} = \frac{2A+B}{2\sqrt{2}}\begin{bmatrix} 1 \\ -i \end{bmatrix}$, and converted parts: $\frac{B}{2\sqrt{2}}e^{-i2\theta}\begin{bmatrix} 1 \\ i \end{bmatrix}$. The non-converted part has the same polarization state as that of the incident beam, while the converted part has an opposite helicity and an additional phase shift -2θ generated by the geometric metasurface. By controlling the orientation angle θ of the nanorod in each unit cell, the phase shift can be tuned, ranging from 0 to 2π.

3.1 Filtering out the non-converted part from the incident RCP light beam

To filter out the non-converted part, a pair of a quarter waveplate (QWP_2) and a linear polarizer (LP_2) is used. The Jones matrix of the QWP_2 is the same as the Jones matrix of QWP_1, but the fast axis is defined as χ_2. The Jones matrix of the LP_2 (J_{P2}) is given as:

$$J_{P2} = \begin{bmatrix} \cos^2\delta & \sin\delta\cos\delta \\ \sin\delta\cos\delta & \sin^2\delta \end{bmatrix}. \quad \text{(S11)}$$

where δ is an angle of the transmission axis of the LP_2 with respect to the x axis. In this case, the angle χ_2 is set parallel to χ_1, which is $\frac{\pi}{4}$. The angle δ is set to 0 which is parallel to LP_1. After the non-converted part (E_{nc}) passing through the LP_2, it can be filtered out as follows:

$$J_{P2}J_{QWP2}\frac{2A+B}{2\sqrt{2}}\begin{bmatrix} 1 \\ -i \end{bmatrix} = \begin{bmatrix} 0 \\ 0 \end{bmatrix}. \tag{S12}$$

After the converted part (E_c) passes through the LP_2, the output vector can be written as

$$J_{P2}J_{QWP2}\frac{B}{2\sqrt{2}}e^{-i2\theta}\begin{bmatrix} 1 \\ i \end{bmatrix} = \frac{B}{2}e^{-i2\theta}\begin{bmatrix} 1 \\ 0 \end{bmatrix}. \tag{S13}$$

Here, the non-converted part is filtered out and the output vector is solely related to the geometric phase, $-2\theta,$ for the incident RCP light beam.

3.2 Incident linearly polarized (LP) light beam

For the incident LP light beam, the QWP_1 and QWP_2 are removed. The Jones matrix of the LP light beam is given by $\begin{bmatrix} \cos\beta \\ \sin\beta \end{bmatrix}$, where β is the inclined angle of the linear polarized direction. β = 0 represents the initial linear polarization direction along the horizontal direction. When the LP beam generated by a linear polarizer LP_1 passes through a nanorod, the Jones vector can be written as:

$$J_{nanorod}\,LP = \frac{2A+B}{2}\cdot\begin{bmatrix} \cos\beta \\ \sin\beta \end{bmatrix} + \frac{B}{2}\begin{bmatrix} \cos\,(2\theta-\beta) \\ \sin\,(2\theta-\beta) \end{bmatrix}. \tag{S14}$$

There are two components emitted from the nanorod, a non-converted part $E_{nclp} = \frac{2A+B}{2}\cdot\begin{bmatrix} \cos\beta \\ \sin\beta \end{bmatrix}$ and a converted part $E_{clp} = \frac{B}{2}\begin{bmatrix} \cos\,(2\theta-\beta) \\ \sin\,(2\theta-\beta) \end{bmatrix}$.

3.3 Filtering out the non-converted part from the incident LP light beam

After the non-converted part (E_{nclp}) passing through the polarizer LP_2, the output vector can be written as:

$$E_{nclp}^{out} = J_{P2}E_{nclp} = \begin{bmatrix} cos^2\delta & sin\,\delta\,cos\,\delta \\ sin\,\delta\,cos\,\delta & sin^2\,\delta \end{bmatrix} \frac{2A+B}{2} \cdot \begin{bmatrix} \cos\beta \\ \sin\beta \end{bmatrix}. \quad \text{(S15)}$$

To filter out the E_{nclp}, the angle δ must be perpendicular to the angle β. Thus, the δ can be $\beta \pm \frac{\pi}{2}$. Therefore, the non-converted part can be filtered out as follows:

$$E_{nc\mathrm{lp}}^{out} = \frac{2A+B}{4}\left\{\begin{bmatrix} \cos\beta \\ \sin\beta \end{bmatrix} + \begin{bmatrix} \cos\,(\beta \pm \pi) \\ \sin\,(\beta \pm \pi) \end{bmatrix}\right\} = \begin{bmatrix} 0 \\ 0 \end{bmatrix}. \quad \text{(S16)}$$

At this moment, the output vector of the converted part passing through the analyzer can be written as:

$$E_{clp}^{out} = J_{P2}E_{clp} = \frac{B}{4}\left\{\begin{bmatrix} \cos\,(2\theta - \beta) \\ \sin\,(2\theta - \beta) \end{bmatrix} + \begin{bmatrix} -\cos\,(3\beta - 2\theta) \\ -\sin\,(3\beta - 2\theta) \end{bmatrix}\right\}. \quad \text{(S17)}$$

According to the predesigned polarization angle ϕ, which is relative to the phase shift of 2θ by the geometric phase. Thus, the Eq. (S17) can be modified as follows:

$$E_{clp}^{out} = \frac{B}{4}\left\{\begin{bmatrix} \cos\,(\phi - \beta) \\ \sin\,(\phi - \beta) \end{bmatrix} + \begin{bmatrix} -\cos\,(3\beta - \phi) \\ -\sin\,(3\beta - \phi) \end{bmatrix}\right\}. \quad \text{(S18)}$$

From Eq. (S18), the minimum intensity captured by a CCD camera can be found at the position that $\phi - \beta = 3\beta - \phi$. Thus, the minimum intensity or a dark gap of the 3D knots can be found at the generated polarization angle $\phi = 2\beta$.

Supplementary Section 4. Creating two 2-foil knots ($\boldsymbol{\phi_{m,n}} = \boldsymbol{\alpha_{m,n}}$) with two operation wavelengths of 650 nm (red) and 580 nm (green)

4.1 The metadevice is illuminated by RCP light beams

To supplement the design presented in **Figure 2**, more simulated and measured intensity distributions at different observation planes (*z* = 390 μm, 400 μm and 410 μm for Knot 1; *z* = 790 μm, 800 μm and 810 μm for Knot 2) under the illumination of RCP light beams at the wavelengths of 650 nm (red) and 580 nm (green) are presented in **Figure S2**. The green Knot

1 can be clearly found at the region between z = 390 μm and 410 μm. The red Knot 2 can be clearly found at the region between z = 790 μm and 810 μm.

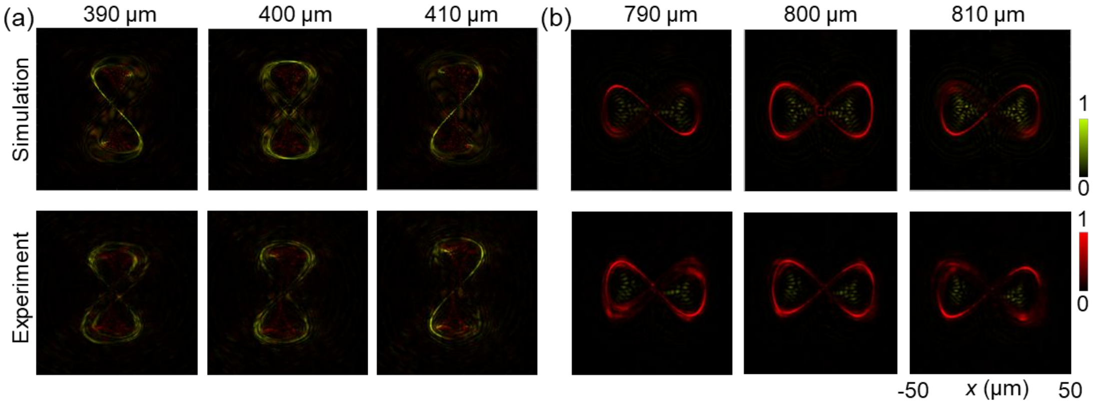

Figure S2. Two 2-foil knots with different colors in a row. The simulated and measured intensity patterns of a) Knot 1 and b) Knot 2 at different observation planes under the illumination of RCP light at two wavelengths of 650 nm and 580 nm.

4.2 The metadevice is illuminated by LP light beams

When the metadevice is illuminated by LP light beams at the wavelengths of 650 nm (red) and 580 nm (green), the simulated and measured intensity distributions of created two 2-foil polarization knots with different colors at six observation planes (z = 390 μm, 400 μm, 410 μm, 790 μm, 800 μm and 810 μm) are shown in **Figure S3**. The transmission axes of the LP_1 and LP_2 are set as (0, π/2) in **Figure S3a** and **Figure S3b** and (π/4, 3π/4) in **Figure S3c** and **Figure S3d**. The dark gaps on the green Knot 1 (**Figure S3a** and **Figure S3c**) and red Knot 2 (**Figure S3b** and **Figure S3d**) can be seen at the predesigned positions with polarization rotation angles ϕ of 0 (π) in **Figure S3a** and **Figure S3b** and π/2 (3π/2) in **Figure S3c** and **Figure S3d**.

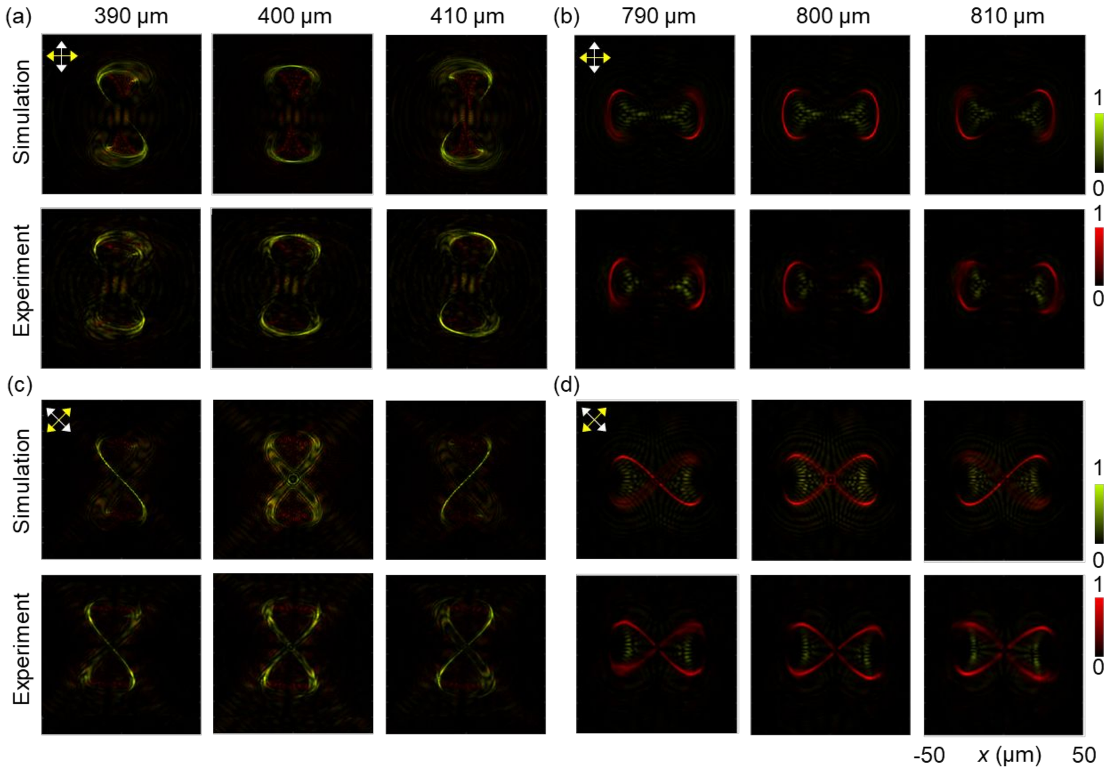

Figure S3. Two 2-foil knots with different colors and polarization profiles. The intensity distributions of 3D polarization structures under the illumination of an incident light beam with inclined linear polarization angles equal to (a, b) 0 and (c, d) π/4 at two wavelengths of 650 nm and 580 nm. Yellow arrows represent the direction of incident polarization through input linear polarizer LP_1, and white arrows show the direction of the output analyzer LP_2.

Supplementary Section 5. Three 2-foil polarization knots ($\phi_{m,n} \neq \alpha_{m,n}$), each knot encoded with two wavelengths

Figure S4 shows the simulation (left) and experimental (right) intensity distributions of the designed three multi-colored 3D knots with nonlinear relation between ϕ and α residing at nine different observation planes (z = 290 μm, 300 μm, 310 μm, 590 μm, 600 μm, 610 μm, 890 μm, 900 μm, and 910 μm). The numbers of focal points N on the Knots 1-3 are 1000, 750 and 600, respectively. The focal points ranging from 1 to 500 on Knot 1 and focal points 1 to 375

on Knot 2 are encoded with a wavelength of 650 nm, where the polarization rotation distribution is set as $\phi_{1,n} = \phi_{2,n} = 0$. The focal points 501 to 1000 on Knot 1 and focal points 301 to 600 on Knot 3, they are encoded with a wavelength of 500 nm, where the predesigned polarization profile is set as $\phi_{1,n} = \phi_{3,n} = \pi/2$. Finally, the focal points ranging from 376 to 750 on Knot 2 and focal points 1 to 300 on Knot 3 are encoded with the wavelength of 580 nm, where the polarization rotation distribution is set as $\phi_{2,n} = \phi_{3,n} = \pi/4$. The results of **Figure S4** are obtained under the illumination of an LP light beam with horizontal polarization direction ($\beta = \pi/8$). The experimental measurements clearly reveal that yellow foils of Knots 2 and 3 with $\phi = \pi/4$ (**Figure S4b** and **Figure S4c**) disappear, which are consistent with the simulation results. Furthermore, it is important to highlight that the intensity distributions exhibit noticeable variations across different observation planes for each knot, further verifying their inherent 3D nature.

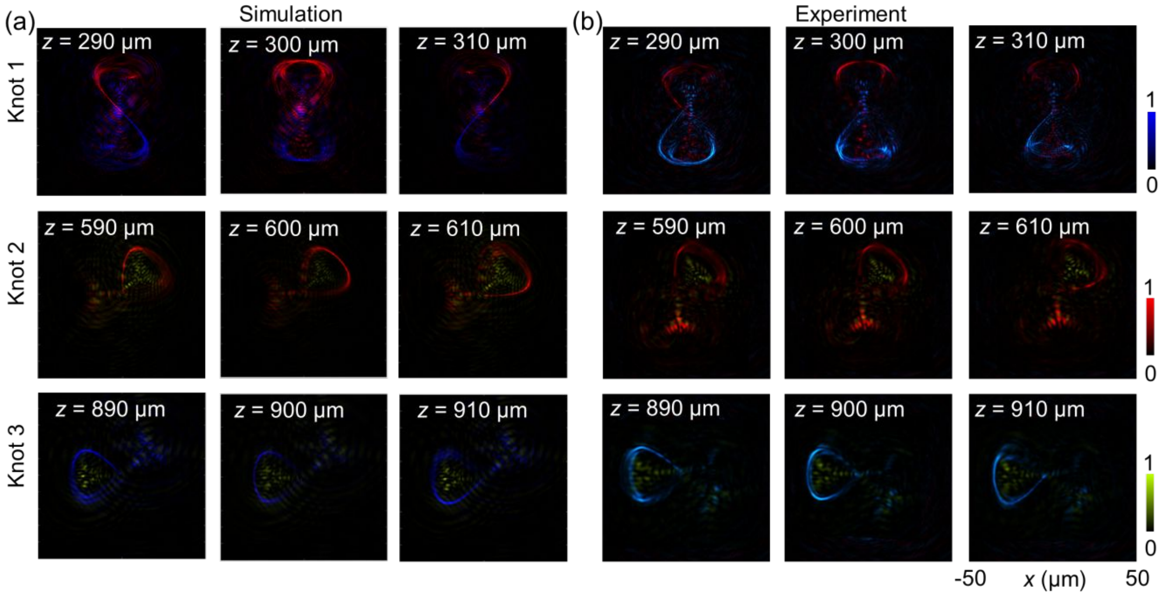

Figure S4. Three multi-colored 3D polarization knots and polarization profiles. Simulated (a) and experimental (b) intensity profiles of Knots 1-3 upon the illumination of incident light beam with an inclined linear polarization angle of $\beta = \pi/8$.

Supplementary Section 6. longitudinally variable 3D color image steganography

In order to show the applicability of proposed design for longitudinally variable 3D color image steganography, we design two 2-foil knots with two colors (**Figure 6**). Specifically, the Knot 1 (m = 1) is encoded with wavelengths of 650 nm and 580 nm, whereas the Knot 2 (m = 2) are designed with wavelengths of 650 nm and 500 nm. The polarization rotation angles for Knot 1 are set as ϕ_{1n} = 0 for $|y_{1n}|$ < 15 μm, and ϕ_{1n} = π/2 for $|y_{1n}|$ ≥ 15 μm. Regarding Knot 2, polarization rotation angles are set as ϕ_{2n} = 0 for $|x_{2n}|$ < 15 μm and ϕ_{2n} = π/2 for $|x_{2n}|$ ≥ 15 μm. The whole 3D cover image is displayed as public information with multiple concealed 3D information. For simulated (top) and measured (bottom) intensity patterns of 3D cover image at six different observation planes (z = 390 μm, 400 μm, 410 μm, 790 μm, 800 μm and 810 μm) are presented in **Figure S5**. The 3D nature of the cover images in the experimental measurement are clearly observed and agree well with the simulation results.

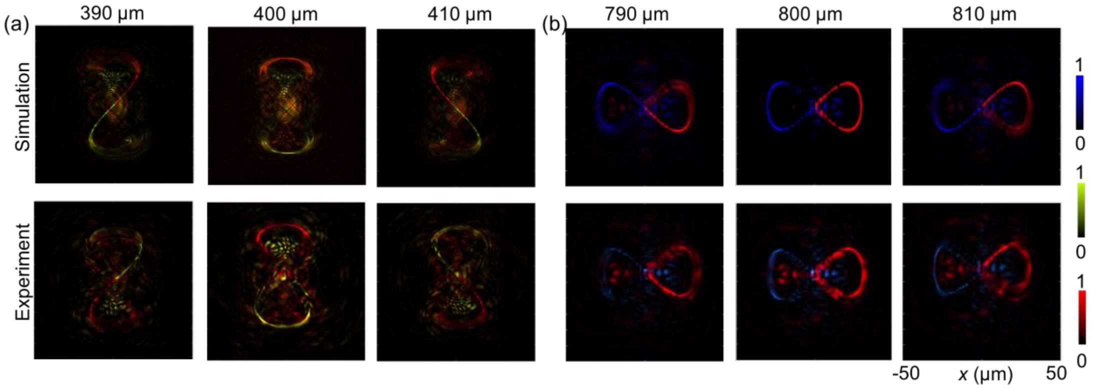

Figure S5. 3D Cover images for longitudinally variable 3D color image steganography. The 3D cover images are treated as public information and can be observed without the knowledge polarization state of the light beam in 3D space.

Figure S6 shows the simulated (top) and measured (bottom) intensity patterns of the concealed 3D information 1, 2, 3 and 4. The desired concealed 3D information can be revealed at the predesigned observation areas under the illumination of an incident LP light beam at predesigned wavelengths and with correct transmission axes of the analyzer (white arrows in **Figure S6**). Therefore, different combinations of the transmission axis of the analyzer, incident wavelengths and the longitudinal-position (z) can function as different keys to reveal multiple distinct concealed 3D information. For example, concealed 3D information 1 and 3 can be obtained with correct key combination i.e., longitudinal positions (z = 390 μm to 410 μm), incident wavelengths (λ = 650 nm and 580 nm) and the transmission direction of the analyzer (π/2 for concealed information 1 and 3π/4 for concealed information 2). Similarly, 3D concealed information 2 and 4 can be obtained with the correct key combination i.e., longitudinal positions (z = 790 μm to 810 μm), incident wavelengths (λ = 650 nm and 500 nm) and analyzer-direction (π/2 for concealed information 2 and 3π/4 for concealed information 4). This technique can dramatically increase the information capacity for image steganography.

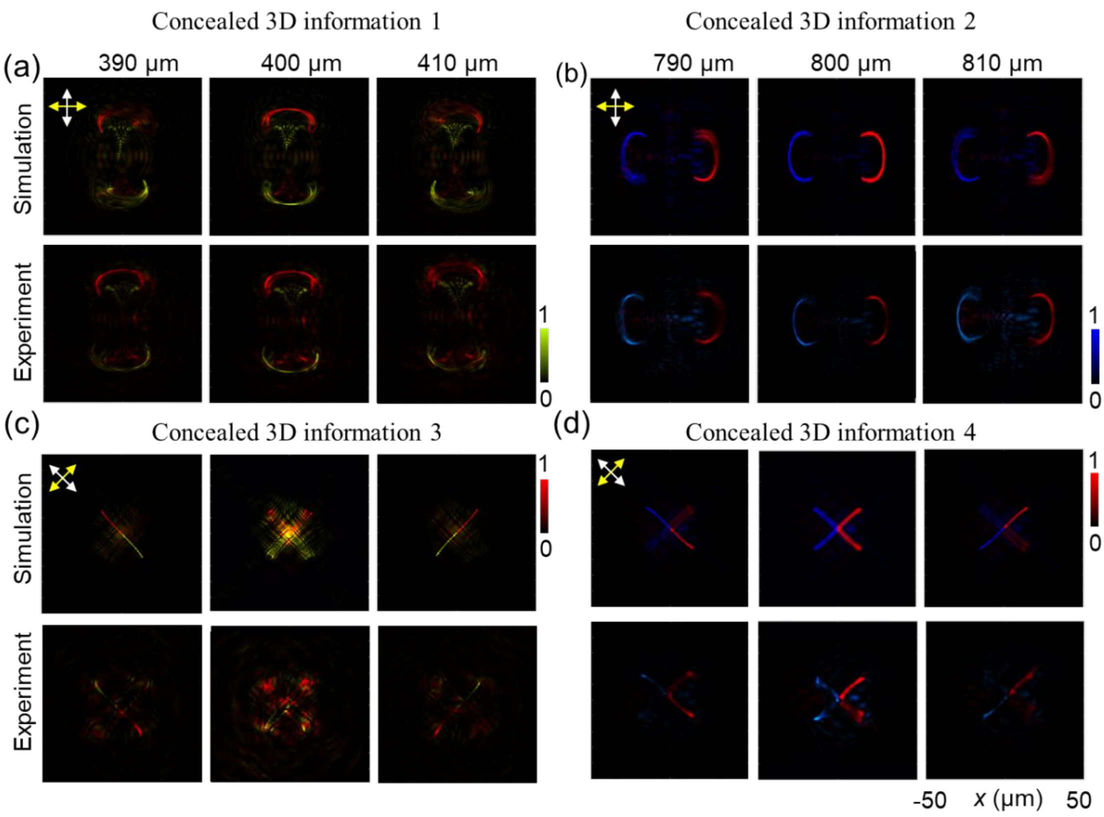

Figure S6. The concealed 3D images within 3D cover images. a) Concealed 3D information 1. b) Concealed 3D information 2. c) Concealed 3D information 3. d) concealed 3D information 4. The desired and concealed 3D information can be revealed with the knowledge of a correct key. For example, concealed 3D information 1 can be obtained with correct key combination i.e., longitudinal positions (z = 390 μm to 410 μm), incident wavelengths (λ = 650 nm and 580 nm) and the transmission direction of the analyzer ($\pi/2$).

Supplementary Section 7. Crosstalk between different colors in the same polarization knot and that between different polarization knots

7.1 Crosstalk between different colors in the same polarization knot

In this work, the idea of the wavelength-encoded design is motivated by the dispersion effect of the metalens, whose focal length changes with the incident wavelengths. To explain the

proposed idea and analyze the crosstalk between different colors in the same polarization knot, we take Knot 1 (**Figure 6**) as an example, which is encoded with two wavelengths (580 nm and 650 nm), and is located at the central observation plane of z = 400 μm, as shown in **Figure S7**. When the metalens is illuminated by light at λ = 580 nm, the encoded green portion of the polarization knot is focused on the predesigned observation region, while another portion of knot is positioned behind this region due to the dispersion effect, generating defocused crosstalk light in the predesigned observation region (**Figure S7**, top). When the incident wavelength is changed to 650 nm, the encoded red portion of knot appears in the observation region, while another portion is away from this area and generating crosstalk (**Figure S7**, bottom). The crosstalk will decrease as the encoded wavelength interval increases due to the more pronounced dispersion.

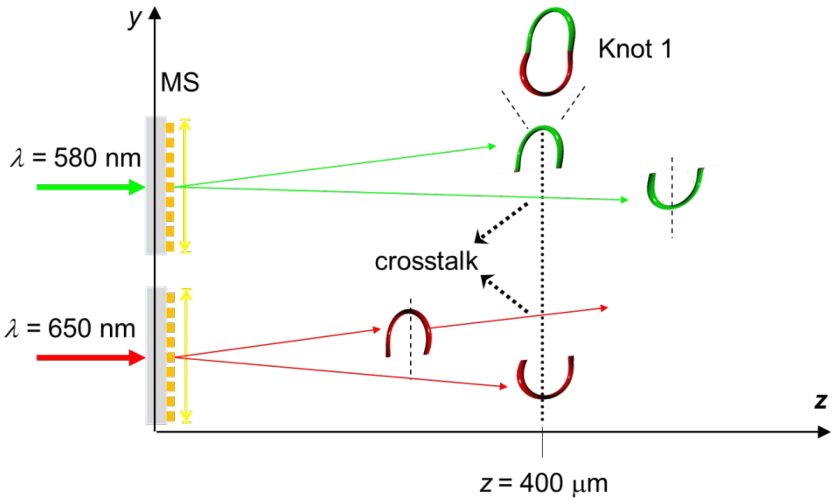

Figure S7. Color-encoded mechanism and the crosstalk between different colors in the same polarization knot. The locations of two different color portions of 3D knot are given upon the illumination of the incident light at λ = 580 nm (top) and λ = 650 nm (bottom), respectively. The observation region is defined by the central observation plane of z = 400 μm.

7.2 Crosstalk between two polarization knots along light propagation direction

In this section, we further analyze the crosstalk between two knots along light propagation direction. As shown in **Figure S8**, the light from the Zones 1-3 (Zone AD) on the metasurface may generate the crosstalk between the two knots. Additionally, at any point within Zone 1 or Zone 3, only light within the angles θ_1 (the angle between rays Y_1E and Y_1H) or θ_3 (the angle between rays Y_3G and Y_3F) will cause mutual crosstalk between two knots. If the number of focal points on Knot 1 and Knot 2 is the same, the energy density of the crosstalk light is evidently greater for Knot 1 (**Figure S8**, top). In Zone 2 (**Figure S8**, bottom), only the light originating from Knot 1 and within the angle θ_2 (the angle between rays Y_2G and Y_2H) cause crosstalk to Knot 2, while all the light from Knot 2 causes crosstalk to Knot 1. Therefore, the crosstalk for the knot at z = 400 μm is stronger than that at z = 800 μm. To reduce the crosstalk at Knot 1, we can appropriately reduce the longitudinal size of Knot 1 and the number of focal points on Knot 2. However, to keep 3D Knot 2 continuous, 700 focal points are used in our design. Another way to improve the image quality is to increase the sample size, as light from regions outside of Zone AD on the metasurface will not cause mutual crosstalk between two polarization knots but will increase the intensity of each knot.

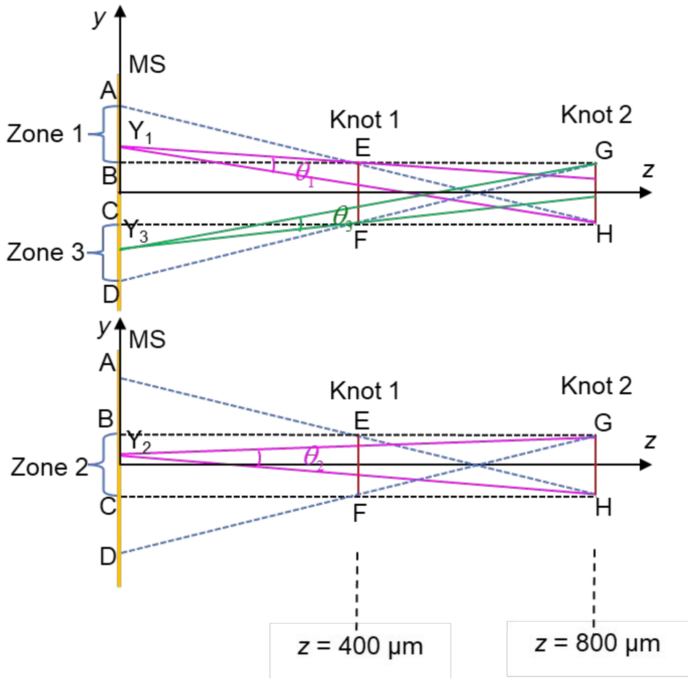

Figure S8. Schematic of crosstalk between two polarization knots along light propagation direction.

Supplementary Section 8. Effect of the encoded wavelength interval on the intensity patterns of each color polarization knot.

Figure S9 shows the simulated intensity distributions of two 2-color 3D knots with the same wavelength-encoded interval (150 µm) for Knot 2, but different intervals for Knot 1: 70 µm in **Figure S9a** and 150 µm in **Figure S9b**. The results are simulated based on the incident RCP light at a single operating wavelength. For the sake of comparison, we select the same design for the 3D polarization knots in **Figure S9a** and **Figure 6**. **Figure S9** (left) clearly shows that the crosstalk at the Knot 1 can be suppressed, and the image quality of the intensity patterns is significantly improved by increasing its encoded wavelength interval. Since the wavelength interval encoded on Knot 2 remains unchanged, the image quality of Knot 2 shows little variation.

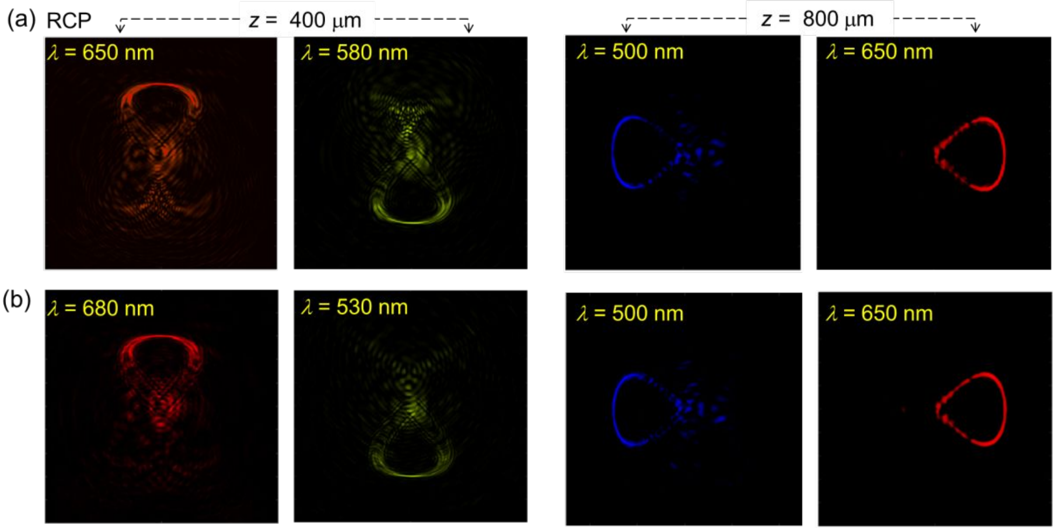

Figure S9. Effect of the encoded wavelength interval of the polarization knot on the intensity patterns at different observation planes located at z = 400 μm and z = 800 μm, respectively. Under the illumination of RCP light at a single operating wavelength, the simulated intensity patterns of two 2-color knots with different encoded wavelength intervals for Knot 1: (a) 70 μm, (b) 150 μm.

Supplementary Section 9. Effect of the longitudinal size of 3D knot and metasurface size on the intensity patterns.

Figure S10 shows the simulated intensity distributions of two two-color 3D knots with different metasurface sizes i.e., 399×399 μm^2 (**Figure S10a**), and 600×600 μm^2 (**Figure S10b**) at the central observation planes z = 400 μm, and z = 800 μm, respectively. The results are simulated based on the incident RCP light at a single operating wavelength. For the sake of comparison, we select the same design for the 3D polarization knots in **Figure S10a** and **Figure 6**. Here, the longitudinal sizes of Knot 1 in **Figure S10a and S10b** are set to S_z = 20 μm (**Figure S10a**) and S_z = 12 μm (**Figure S10b**), respectively. In **Figure S10**, we notice that by increasing the area of the metasurface to 600×600 μm^2 and decreasing the longitudinal

size of Knot 1, the parasitic light (especially for Knot 1) is effectively suppressed, and the better quality of intensity patterns of two-color polarization knots is achieved.

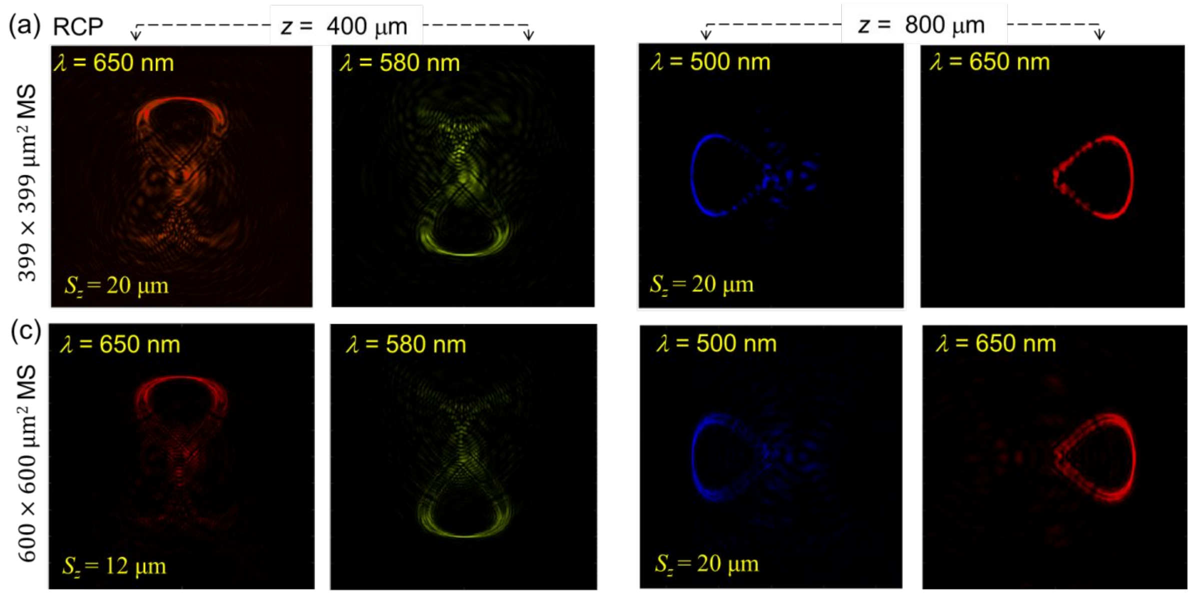

Figure S10. Effect of longitudinal size (S_z) of 3D knot and metasurface size on the intensity patterns. Simulated intensity patterns of two 2-color knots located at z = 400 μm and z = 800 μm under the illumination of RCP light at a single operating wavelength. (a) The area of the metasurface is 399×399 μm^2. The longitudinal size of both Knot 1 and Knot 2 is set to S_z = 20 μm. (b) The area of the metasurface is 600×600 μm^2. The longitudinal sizes of Knots 1 and 2 are set to S_z = 12 μm and S_z = 20 μm, respectively.

Supplementary Section 10. Creating two 2-foil knots ($\phi_{m,n} \neq \alpha_{m,n}$) with three colors along the longitudinal direction

In this section, metasurface with two 2-foil knots with three colors and unique polarization rotation angles is designed. The encoded wavelengths and polarization rotation angles for two knots are $\lambda = 650$ nm and $\phi = 0$ for $\alpha \in [0, 2\pi/3)$, $\lambda = 580$ and $\phi = \pi/4$ for $\alpha \in [2\pi/3, 4\pi/3)$, and $\lambda = 500$ and $\phi = \pi/2$ for $\alpha \in [4\pi/3, 2\pi)$. The simulated intensity distributions

at different central observation planes (z = 400 μm for Knot 1; z = 800 μm for Knot 2) under the three-wavelength illumination with different polarization states are presented in **Figure S11**. Two 2-foil knots with three colors are clearly shown for the incident RCP light beams at the wavelengths of 650 nm, 580 nm and 500 nm (Column 1 in **Figure S11**). For the incident LP light beams with the polarized direction angles β = 0, π/8, and π/4 (yellow arrows in **Figure S11**), the gaps can be found at the positions where the predesigned polarization rotation angles ϕ = 2β and 2β+π (Columns 2-4 in **Figure S11)**.

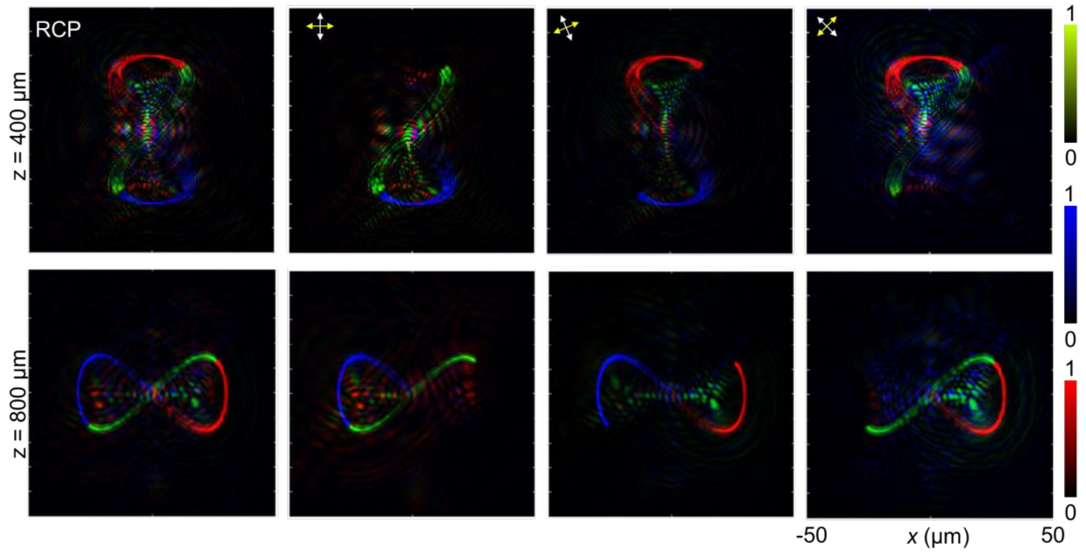

Figure S11. Two 2-foil knots with three colors and polarization profiles. The simulated intensity profiles of two 2-foil knots under the three-wavelength illumination (λ = 650 nm, 580 nm and 500 nm) with different polarization states (RCP, and LP with the polarized direction angles β = 0, π/8, and π/4 (yellow arrows)).
